# A comprehensive perspective on electric vehicles as evolutionary robots

**DOI:** 10.3389/frobt.2025.1499215

**Published:** 2025-02-17

**Authors:** Haoyang Che, Shaolin Wang, Lei Yao, Ying Gu

**Affiliations:** ^1^ User Digitization Department, Zeekr Group, Hangzhou, China; ^2^ Zhejiang Automotive Engineering Institute, Geely Holding Group, Hangzhou, China

**Keywords:** EV, evolutionary robots, software-defined vehicles, AI-defined vehicles, AI agent

## Abstract

Multi-robot systems exhibit different application forms in human life, among these, electric vehicles (EVs) at rest and in motion can be perceived as a specialized category of multi-robot systems with increasingly sophisticated vehicle functions and a certain degree of flexibility, and most notably, the ability to iteratively evolve. However, for EVs to evolve into the next-generation of multi-robot systems, more complex technical and operational mechanisms shall be fully cultivated in EVs to develop their evolutionary capabilities, including, but not limited to multimodal environmental sensing techniques, advanced telematics communication protocols such as 5G, Over-The-Air (OTA) upgrade functions, real-time backend data lake analytics, and user-centric marketing initiatives. As it stands, these mechanisms are evidently insufficient for realizing genuine evolutionary robots (ER), especially in unstructured environments. The overarching perspective of conceptualizing EV as ER is not always prominently featured in academic literature. This manuscript provides a succinct overview of the ongoing transition from Software-Defined Vehicles (SDV) to Artificial Intelligence-Defined Vehicles (AIDV), and examines the ongoing research focused on the utilization of electric vehicles as mobile edge computing platforms. Furthermore, it discusses the fundamental evolutionary competencies that define modern electric vehicles, establishing the core tenets upon which our analysis is predicated. To transcend the *status quo*, we underscore the imperative and pressing need for profound transformations across a spectrum of pivotal domains within the field. Furthermore, this endeavor aims to amplify the reach and influence of research on EVs as ERs, potentially catalyzing the emergence of several niche research areas.

## 1 Introduction

Over the past decade or so, the field of evolutionary robotics has garnered significant attention from a diverse cohort of researchers, spanning disciplines such as artificial intelligence, robotics, biology, cognitive science, and even social behavioral studies. Almost at the same time, electric vehicles have also experienced rapid growth and disruptive advancements, evolving from the conceptualization of Domain Control Unit (DCU) (Müller-Glaser, 2006), to a more comprehensive understanding of Electrical/Electronic Architecture (EEA) (Zhu et al., 2021). The evolution continues from the initial coining of SDV (Bodei et al., 2023), to the more recent advocacy of AIDV (Wu, 2024).

Although the convergence of these two previously distinct domains is seldom highlighted in academic literature, there is a growing trend, with a substantial amount of integration activities stemming from the particularly dynamic automotive sector. Despite the academic community’s relative lagging compared to industry advancements, it is poised to closely follow this trend. The academic community will engage in a series of pertinent theoretical investigations aimed at bolstering practical applications and ensuring that theoretical insights are effectively translated into real-world benefits.

Within the EV industry, robotic systems have been extensively implemented for a multitude of applications. For instance, the proliferation of electric vehicles has led to a corresponding surge in charging demands over recent years. To mitigate this challenge, several companies have engineered autonomous charging robots capable of replenishing the energy of electric vehicles, thereby introducing an innovative charging paradigm. A case in point is Volkswagen’s introduction of the Mobiler Laderoboter, a mobile charging robot that employs a robotic arm and integrated batteries to service electric vehicles (Volkswagen Newsroom, 2024). Beyond the deployment of charging robots, the realm of battery recycling for electric vehicles represents another critical area intersecting with robotics technology. As the EV fleet expands, the management and recycling of spent batteries have become increasingly pertinent. Researchers are currently developing a knowledge graph that empowers robots to disassemble discarded EV batteries, thereby enhancing the efficiency and safety of the recycling process (Wang et al., 2023).

In the manufacturing arena, robotic technology is pervasive in the automated production lines of electric vehicles, where it plays a pivotal role in augmenting production efficiency and quality. A notable example is the fully automated electronic component production line developed by SAR company, which utilizes KUKA robots to manufacture key components for electric vehicles (KUKA, 2024). The interplay between electric vehicles and robotics is one of synergy and mutual enhancement. We posit that electric vehicles, while visually distinct from humanoid robots such as Tesla’s Optimus (Ali et al., 2023), are increasingly embodying the characteristics of evolutionary robots. This evolving relationship underscores the potential for electric vehicles to transcend traditional automotive boundaries and integrate advanced robotic functionalities, heralding a new era of intelligent, adaptive transportation.

Throughout the entire use phase of an electric vehicle, the vehicle undergoes continuous evolution. In contrast to traditional internal combustion engine vehicles, which are delivered to users in one shot, EVs are equipped with pre-embedded hardware that can be remotely activated. OTA updates continuously refine the software and data profiles of these vehicles (Halder et al., 2019). Additionally, the autonomous driving perception systems of EVs continuously monitor the environment both inside and outside the vehicle (Hu et al., 2024; Cheng et al., 2021; Cheng et al., 2024). Consequently, the status of an EV can differ from 1 minute to the next, whether the vehicle is in motion or at rest. This dynamic nature underscores the continuously evolving characteristic of electric vehicles.

We assert with conviction that the current phase is embryonic in the evolution of EVs into sophisticated evolutionary robots. This perspective aligns with the burgeoning capabilities of EVs to adapt and improve through software updates and environmental interactions, foreshadowing a transformative shift towards vehicles that are both environmentally sustainable and technologically adaptive.

In this context, we articulate two distinct yet interrelated viewpoints on the evolution of EVs towards evolutionary robots:

1. The continuous progression of OTA updates, coupled with the acquisition of environmental information by perception systems and the dynamic changes in vehicle status attributable to vehicular components such as internal power and transmission systems, will drive the relentless evolution of electric vehicles. This progression is anticipated to culminate in the emergence of electric vehicles as sophisticated evolutionary robots.
2. Owing to the relentless advancement of the Internet of Vehicles (IoV), a networked ecosystem of multiple electric vehicles is poised for perpetual evolution. This development is projected to culminate in the formation of an advanced evolutionary multi-robot system, underscoring the convergence of vehicular technology with robotics and Internet of Things (IoT) infrastructure.

**FIGURE 1:**
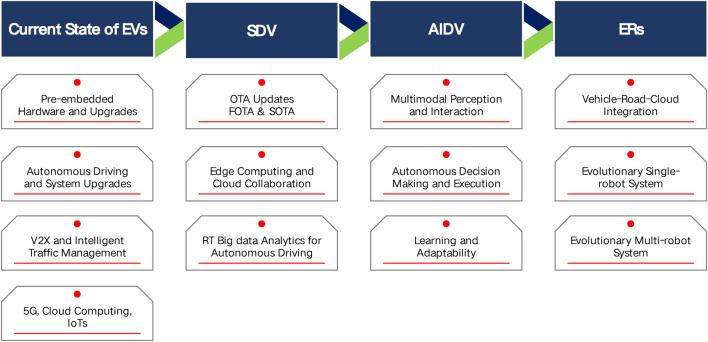
Development Path of EVs towards ERs. It is posited that the evolutionary trajectory of electric vehicles will progress through distinct phases, beginning with SDV, advancing through AIDV, and ultimately leading to the emergence of individual evolutionary vehicle robots. This progression will culminate in the sophisticated formation of an integrated evolutionary multi-robot system, marking a significant leap in the convergence of vehicle technology and robotics.

In light of the above perspectives, Figure 1 delineates the pivotal stages in the transformation of electric vehicles into evolutionary robots:

These industry advancements underscore the significance of our research, which posits that EVs are not only becoming increasingly similar to evolutionary robots in terms of on-vehicle functions and processing performance but are also poised to actualize this potential within the foreseeable future, perhaps within the next decade. To substantiate this hypothesis, Section 2 initially delineates the paradigm shift from SDV to AIDV, which serves as a pivotal catalyst in propelling the evolution of EVs into sophisticated evolutionary robots. Notably, this evolution is not merely theoretical; it is being actively pursued by industry leaders. Section 3 examines the computational paradigm of cloud-edge collaboration, within which electric vehicles are envisioned to function as mobile edge computing nodes. This paradigm not only facilitates the computational demands of EVs but also lays the groundwork for their evolutionary paths. The current models of electric vehicles have already integrated certain evolutionary capabilities, including pre-embedded hardware mechanisms, OTA updates, and the perceptual subsystems of autonomous driving systems, as described in Section 4. While current EVs possess fundamental evolutionary attributes requisite for robotic functions, a significant disparity remains when juxtaposed with prospective intelligent vehicle models, as detailed in Section 5. In the final analysis, Section 6 offers a concise discussion on the aforementioned perspective and provides a conclusive synthesis of the paper’s findings.

## 2 From SDV to AIDV

**FIGURE 2:**
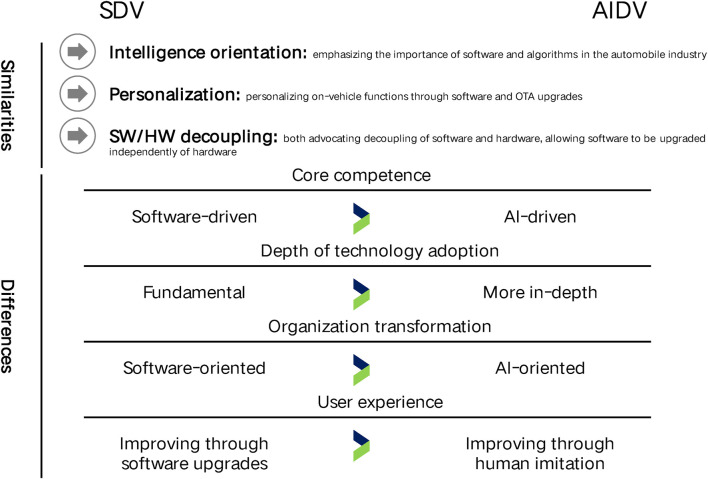
Transition from SDV to AIDV. Within the automotive industry’s trajectory from SDV to AIDV, both paradigms exhibit a multitude of similarities and distinctions. This transition encapsulates a complex interplay of technological evolution and strategic adaptation, highlighting the dynamic nature of innovation in the field of vehicular development.

In recent years, a seminal development within the EV sector has been the paradigm shift from SDV to AIDV, as illustrated in Figure 2. This transition encapsulates the evolution of EVs from hardware-centric to software-centric platforms, with SDV advocating for the decoupling of vehicular software from hardware development, thereby unlocking the evolutionary potential of EVs (Arole, 2023). AIDV represents an intensification of this concept, integrating artificial intelligence (AI) to further enhance the capabilities of EVs.

The United States and China have emerged as proactive participants and leaders in the SDV era, driving innovation and setting industry standards. This development is bolstered by the global automotive industry’s commitment to SDV infrastructure. Furthermore, Tesla’s pioneering role in SDV is evident through its introduction of OTA updates and autonomous driving technologies, which have become foundational to the ongoing evolution of EVs.

Jensen Huang, CEO of NVIDIA, has envisioned a future where software not only defines vehicles but also generates revenue, stating that the business model of car manufacturers will fundamentally change. NVIDIA has partnered with Mercedes-Benz to integrate AI and metaverse technologies into its design and development process, utilizing NVIDIA DRIVE Orin™ to create software-defined vehicles and NVIDIA Omniverse™ for more intelligent and efficient manufacturing—a testament to the practical application of these concepts (NVIDIA Blog, 2024).

Volkswagen, another proponent of SDV, maintains that software will be pivotal in achieving competitive differentiation in the 21st-century automotive market (Volkswagen Newsroom, 2021). These industry advancements underscore the significance of SDV and AIDV in shaping the future of the automotive landscape, where software and AI are poised to redefine the essence of mobility.

Undoubtedly, China has emerged as the world’s largest and most rapidly expanding EV market, characterized by swift transformations and intense competition. The market landscape is populated by a diverse array of players, including established automotive conglomerates, EV specialists, telecommunications corporations such as Huawei, independent chip manufacturers like Horizon Robotics (HR, 2024) and Black Sesame (BS, 2024), innovative startups, and academic research communities.

XPENG, a vanguard in China’s EV sector, has inked a Master Agreement with Volkswagen to accelerate the joint development of two intelligent electric vehicles for the Volkswagen brand. This collaboration is projected to slash the time-to-market by over 30 percent, underscoring the strategic importance of such alliances in the EV industry (Volkswagen Group, 2024).

Huixiao Wu, CTO of Great Wall Motors (GWM), has articulated at an industry forum that the swift development and widespread adoption of Large Language Models (LLM) herald a new inflection point for the automotive industry. She posits that the industry is on the cusp of transitioning from SDV to AIDV, reflecting a paradigm shift in vehicle development and functionality (GWM, 2024). Echoing the sentiments of NVIDIA and GWM, industry stalwarts such as Changan Automobile, XPENG, and NIO have also proclaimed that the AIDV era has not only arrived but is already reshaping the automotive landscape.

Beyond the United States and China, several European and Asian countries have demonstrated significant advancements in the EV sector. Notably, NXP Semiconductors, a leader in automotive processing, supports the evolution of domain control and zonal control within SDV architectures, emphasizing their commitment to electrification and vehicle autonomy (NXP, 2024). Looking ahead, Toyota’s planned rollout of its vehicle OS, Arene, in 2025, signifies the Japanese automaker’s strategic entry into the SDV market (Toyota, 2024).

SDV and AIDV are central to the evolution of EVs into sophisticated evolutionary robots. SDV enables the software and data capabilities necessary for evolution by decoupling software development from hardware, facilitating advanced data analytics and OTA updates. AIDV advances this evolution by integrating AI capabilities, which are critical for autonomous decision-making and real-time environmental adaptation. The integration of SDV and AIDV is essential for creating EVs that can evolve and adapt, leading to a new era of intelligent, self-improving vehicles. As these vehicles become integral components of smart infrastructure and IoT networks, they are poised to revolutionize the automotive sector and influence urban development and environmental strategies.

## 3 Electric vehicles as mobile edge computing devices

As detailed in Section 2, the paradigm of SDV has increasingly gained traction in the automotive industry, marking a paradigm shift where vehicles are no longer perceived solely as electromechanical transportation devices but are evolving into mobile edge computing devices or supercomputing terminals with enhanced intelligent and networked capabilities (Xiaolong et al., 2019; Dai et al., 2020). This transformation is pivotal as it positions EVs to leverage computing, storage, and network services in close proximity, thereby reducing latency, improving response times, and alleviating the load on cloud data centers (Yi et al., 2015).

This computing paradigm is particularly significant, as it enables real-time data processing and analysis, underpinning key functionalities such as autonomous driving, Vehicle-to-Vehicle (V2V) communication, and intelligent traffic management. The integration of these capabilities is not only transforming the automotive industry but also laying the groundwork for a new era of smart mobility, where vehicles are integral components of a larger, interconnected ecosystem. This evolution underscores the significance of SDV in facilitating the transition towards a future where vehicles are not just means of transport, but also nodes in a broader network of intelligent, data-driven systems (Lu and Shi, 2024).

Against this backdrop, the current research and applications are primarily concentrated in the following areas:

1. Edge intelligence within the Internet of Vehicles: The focus of this research domain is the migration of AI to the periphery of transportation data sources. By harnessing the computational power, storage resources, and perceptual capabilities of the edge, this area aims to enhance resource allocation and processing mechanisms, thereby delivering smarter and more efficient solutions. This encompasses real-time responsiveness, intelligent decision-making processes, and network autonomy, which collectively contribute to the evolution of the IoV from a mere data transmission network to an intelligent information platform.
2. Collaborative Edge and Cloud Computing in Intelligent Connected Vehicles: The advancement of intelligent connected vehicles necessitates a paradigm of global intelligence, within which edge-cloud collaborative computing emerges as a pivotal trend. This integrated computing model amalgamates the strengths of both edge and cloud computing paradigms. It leverages the swift, localized processing and response capabilities of edge computing, which are essential for safeguarding privacy, while simultaneously harnessing the extensive computational and storage prowess of cloud computing for in-depth data analysis and management. This synergistic approach is crucial for the development of intelligent connected vehicles, enabling a balance between real-time, on-site decision-making and the comprehensive data handling required for complex vehicular operations.
3. Computation Offloading and Resource Allocation in the Internet of Vehicles: Within the IoVs, vehicles often contend with limited computational resources. To address this, these vehicles can offload intensive computational tasks or latency-sensitive operations to edge servers via onboard wireless communication technologies. By tapping into the substantial computational resources of edge servers, vehicles can augment their own capabilities, thereby enhancing the quality of service (QoS) for connected car applications. This approach not only bolsters the performance of vehicular applications but also optimizes the energy consumption and allocation of computing resources within the vehicle. This strategy is essential for managing the trade-offs between on-board processing and the leveraging of external computational power, ensuring that IoV applications meet the stringent requirements of real-time performance and efficiency. (Yi et al., 2019; Dai et al., 2020).
4. Deep Learning Model Training and Inference within the Internet of Vehicles: In the context of the IoVs, the synergy between edge computing and AI is contingent upon the efficiency of model training and inference processes. This domain encompasses a spectrum of training methodologies, including centralized, decentralized, and hybrid approaches. A critical aspect of these methodologies is the development of strategies that not only ensure the effectiveness of model training and application but also safeguard user privacy. The challenge lies in striking a balance between leveraging the distributed nature of IoV data for comprehensive model training and maintaining the confidentiality and integrity of user information. This requires innovative solutions that integrate advanced cryptographic techniques, federated learning, and privacy-preserving machine learning algorithms to facilitate robust AI capabilities while adhering to stringent privacy standards.

In conclusion, the research and applications pertaining to the utilization of electric vehicles as edge computing devices are advancing apace, encompassing the IoVs, edge-cloud collaborative computing, computation offloading, and deep learning model training, among other areas. These endeavors are directed towards enhancing the intelligence quotient and user experience of intelligent electric vehicles. The trajectory of electric vehicle evolution extends beyond the development of individual vehicles; it also encompasses vehicle-to-vehicle co-evolution. As electric vehicles progress towards becoming fully-fledged mobile edge computing devices, their capacity for evolution is expected to become increasingly comprehensive and refined. This transformation promises not only to elevate the capabilities of individual vehicles but also to revolutionize the interconnected ecosystem of smart mobility, underscoring the pivotal role of electric vehicles as integral components of a broader, intelligent transportation network.

## 4 Core evolutionary capacities of current electric vehicles

The core evolutionary capacities of contemporary electric vehicles reflect the convergence of cutting-edge technologies that position them at the vanguard of the automotive industry’s transformation. These capacities are pivotal for the vehicles’ ability to adapt, learn, and evolve over time, akin to living organisms in their responsiveness to environmental stimuli and internal processes. The following are several key areas that encapsulate the core evolutionary capacities of current electric vehicles:

1. Modular Design and Upgradability: The modular design of current EVs allows for the easy replacement and upgrading of components, such as batteries and electronic control units, ensuring that vehicles can evolve with technological advancements.
2. Pre-embedded Hardware: Pre-embedded hardware enables vehicles to be equipped with the necessary components for advanced features such as autonomous driving, infotainment systems, and connectivity, even before the software to fully utilize these components is developed or mature.
3. OTA Updates: The ability to receive OTA updates allows EVs to continuously improve their software, incorporating new features, patches, and performance enhancements without the need for physical intervention.
4. Autonomous Driving Capabilities: With the integration of autonomous driving technologies, EVs can evolve their driving strategies, learn from driving data, and improve their navigation and path-finding algorithms, leading to higher levels of autonomy.
5. Energy Management Systems: Advanced energy management systems in EVs allow for the optimization of battery usage, regenerative braking, and charging strategies, ensuring that the vehicles become more efficient over time.
6. Vehicle-to-Everything (V2X) Communication: V2X communication capabilities enable EVs to interact with other vehicles, infrastructure, and even pedestrians, facilitating a more integrated and responsive transportation ecosystem.
7. Sustainability and Energy Regeneration: As part of their evolutionary journey, EVs are increasingly focusing on sustainability, with capacities for energy regeneration and the use of renewable energy sources for charging, reducing their carbon footprint over time.
8. Data Analytics and Machine Learning: The collection and analysis of driving data allow EVs to apply machine learning techniques for predictive maintenance, improved driving experiences, and the development of personalized features.
9. Adaptive Intelligence: Modern EVs are equipped with sophisticated onboard processing units and AI algorithms that enable them to adapt to varying driving conditions, optimize energy consumption, and enhance safety through real-time decision-making.

Within the realm of SDV, the concepts of pre-embedded hardware and OTA updates are of paramount importance. These paradigms are indicative of a shift where the value and quantity of software and electronic hardware in a vehicle are anticipated to surpass that of mechanical components. Pre-embedded hardware, which consists of advanced electronic components and systems integrated into vehicles prior to their release, is designed to augment vehicle capabilities and facilitate future upgrades and functionalities through software updates. This approach not only expedites the time-to-market for new vehicle models but also establishes a versatile platform for the continuous introduction of new features and enhancements as software development evolves.

OTA updates, which encompass Firmware-Over-The-Air (FOTA) and Software-Over-The-Air (SOTA), refer to the remote upgrading and updating of software or firmware on devices via wireless communication technology. This capability is pivotal in the SDV context, as it enables the seamless integration of new functionalities, performance improvements, and security patches without the need for physical access to the vehicle. Consequently, OTA updates are a cornerstone of the SDV evolution, enabling vehicles to remain at the cutting edge of technology throughout their operational lifecycle and ensuring that they can adapt to emerging requirements and standards.

FOTA: FOTA pertains to the comprehensive upgrading of deeper system layers, encompassing firmware revisions for a vehicle’s core operating system and Electronic Control Units (ECUs). This process is akin to the updating of operating systems on smartphones, where FOTA enables the enhancement of fundamental vehicle components. These updates have the potential to influence critical systems, including the powertrain and safety control mechanisms of the vehicle, thereby necessitating a high level of precision and reliability in the implementation of such firmware upgrades (Arakadakis et al., 2022).
SOTA: SOTA is primarily directed towards the updating of non-critical vehicle applications and functionalities, including infotainment systems, navigation software, and user interfaces. This process is analogous to the routine updating of applications on mobile phones, where SOTA facilitates the seamless enhancement and refinement of these secondary systems without compromising the core operational integrity of the vehicle (Ghosal et al., 2022).

Table 1 outlines the differences between FOTA and SOTA in the context of the automotive industry:

**TABLE 1:** FOTA vs. SOTA. This table highlights the key differences between FOTA and SOTA updates in the automotive industry, emphasizing their distinct roles in vehicle evolution and user experience enhancement.

Criteria	FOTA	SOTA
Update Content	Updates firmware, which includes core system and controller updates such as engine, transmission, and chassis	Updates software, typically involving infotainment systems, navigation maps, and applications
Technical Complexity	High, as it involves changes at the firmware level, which can affect the vehicle’s performance and safety	Lower, as it deals with software applications and user interfaces, not core vehicle functions
Risk Level	Higher due to the potential impact on vehicle control and safety systems	Lower, as software updates are less likely to affect critical vehicle operations
Update Process	May require the vehicle to be stationary and could necessitate a reboot, temporarily affecting vehicle use	Can often be performed while the vehicle is in use, with minimal to no disruption to the user
Provider of Updates	Typically provided by the vehicle manufacturer	May be provided by third-party application developers or suppliers
Purpose of Updates	To improve device performance, functionality, and security through deep system updates	To enhance user experience and interface optimization by providing the latest features and bug fixes
User Impact	Updates can be more intrusive, potentially requiring user downtime and attention during the update process	Updates are designed to be more user-friendly, with minimal impact on the user’s interaction with the vehicle
Advantages	Provides the ability to make significant improvements to vehicle performance and add new features	Allows for rapid iteration of software applications, keeping the user experience fresh and up-to-date

In the evolutionary trajectory of intelligent electric vehicles, the convergence of pre-embedded hardware and OTA updates has emerged as a defining trend. Advanced hardware components are integrated into vehicles with the functionalities and value of these components being progressively activated and enhanced through OTA systems throughout the vehicle’s lifecycle. The synergy between these two technological advancements facilitates the swift iteration of electronic hardware technology, aligning with the protracted development cycles inherent to vehicle production.

Furthermore, Original Equipment Manufacturers (OEMs) can leverage OTA updates to incrementally introduce new features, thereby extending the lifecycle of their vehicles and sustaining product competitiveness amidst fluctuating market demands and technological advancements. These capabilities endow electric vehicles with fundamental evolutionary traits, enabling them to adapt and improve over time.

Additionally, the perceptual hardware units embedded in electric vehicles confer the ability to dynamically alter their operational states. Concurrently, both the vehicles and associated cloud servers accumulate substantial volumes of state data, which is instrumental for the vehicles’ ongoing evolution. This data serves as a cornerstone for continuous improvement, underpinning the vehicles’ capacity to evolve in response to new challenges and opportunities within the dynamic landscape of transportation technology.

Beyond the aforementioned technologies, the presence of autonomous driving perception systems significantly bolsters the evolutionary capabilities of electric vehicles. The primary function of these automated driving perception systems is to detect environmental changes pertinent to driving and to gather pertinent information. This encompasses the suite of sensors and systems unique to autonomous driving, including cameras, millimeter wave radars, lidar, ultrasonic sensors, and high-precision maps, which form the foundation of the autonomous driving system (ADS). Broadly, this definition extends to all sensing units that contribute to the operation of the auto drive system, such as humidity sensors, driver monitoring systems, speed sensors, and torque sensors. These perceptual data feeds are integral to the transition judgments within the ADS state machine and the computation of ADS planning and control directives.

As the sophistication of autonomous driving levels advances, so too does the demand for computational power. In the automotive industry, computing power has emerged as a critical competitive metric, supplanting the traditional emphasis on horsepower. It is thus poised to become a pivotal force in propelling the intelligence and digitization of the automotive sector. Moreover, computing power serves as the cornerstone for the comprehensive evolution of electric vehicles, enabling them to meet the escalating complexities of autonomous operation and to adapt to the evolving landscape of vehicular technology.

These core evolutionary capacities are revolutionizing not just the capabilities of individual EVs, but also the broader landscape of smart mobility and sustainable transportation. With ongoing research and development in these domains, the future of electric vehicles is poised to become more interconnected, intelligent, and adaptable than at any previous point in history. This evolution heralds a new era in transportation, where EVs are not merely vehicles but also nodes in a larger network of intelligent robotic systems, offering unprecedented levels of connectivity, automation, and environmental responsibility.

## 5 Gap between the present and the future

While current EVs exhibit fundamental characteristics and capabilities for dynamic evolution, and advancements in this domain have been swift, there remain several shortcomings and areas for improvement in the full realization of multi-robot systems, particularly within unstructured environments. To encapsulate these considerations, the following points are summarized:.

**FIGURE 3:**
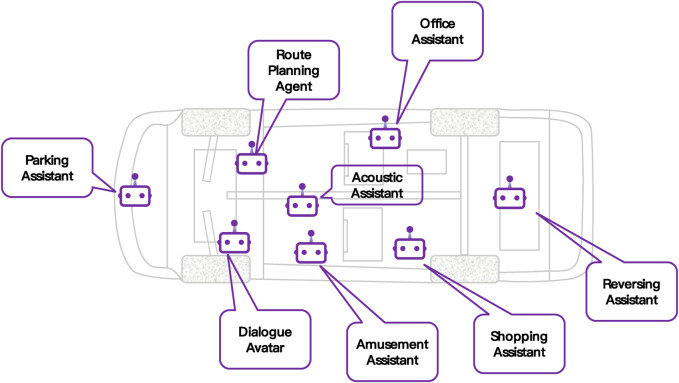
On-Vehicle AI Agents Across Diverse Cabin Scenarios. AI agents, endowed with multimodal perception, interaction, dialogue, planning, and reasoning capabilities, are poised to execute more autonomous decision-making processes and initiate more intelligent actions, all at significantly reduced learning costs. These advancements position AI agents as pivotal components within the evolving landscape of on-vehicle technology, enhancing the operational autonomy and cognitive efficiency of modern electric vehicles.

1. Maturity of Electrical/Electronic Architecture (EEA): The automotive sector is navigating a critical transition from EEA 2.5 to EEA 3.0, a shift that is essential for the incremental enhancement of the evolutionary capabilities inherent in electric vehicles. The stability and sophistication of the EEA version are of utmost importance, as they form the foundation upon which the vehicle’s capacity to assimilate increasingly intricate software and hardware systems rests. This integration is crucial for enabling advanced functionalities and enhancing vehicular performance, thereby driving the evolution of electric vehicles towards greater complexity and autonomy.
2. Success Rate of OTA Updates in EVs: OTA updates, which are integral to the evolutionary capabilities of EVs, have yet to attain a 100% success rate in upgrades, and the duration of these updates remains excessively prolonged. The technology, which is still reliant on extensive support from professional teams within OEMs, has not yet reached a state of optimal maturity. It is evident that further developments are imperative to bolster the reliability and efficiency of OTA updates. Achieving high-speed, fail-safe execution of these updates is essential for the unimpeded advancement of EV technology, ensuring that they can evolve in line with the demands of modern smart mobility and sustainable transportation systems.
3. OTA Updates and Evolution-Related Technologies: The deployment of OTA updates and pre-embedded hardware, along with other upgrade technologies pivotal to the evolution of electric vehicles, may engender numerous security breaches and conflicts with user privacy. It is both imminent and imperative to address these concerns effectively and efficiently to ensure the long-term sustainability and ethical development of these technologies within the automotive industry. The safeguarding of user data and the integrity of vehicle systems must be a cornerstone of technological advancement, necessitating robust security protocols and privacy-preserving measures to mitigate potential risks and maintain public trust.
4. Computing Power Scale and Evolutionary Capability: The scale of computing power serves as a critical determinant of the evolutionary capability of electric vehicles, dictating both the performance and velocity of their evolutionary progress. Currently, the computing power of electric vehicles, when considered as edge computing servers, is in a phase of ongoing enhancement and remains far from reaching its ultimate potential. This ongoing development underscores the need for continuous advancements in computational capacity to support the complex and dynamic evolution of electric vehicles within the emerging landscape of smart mobility.
5. Evolution-Related Systems and Real-Time Processing: Numerous systems pivotal to the evolutionary capabilities of electric vehicles, including perception, decision-making, and execution components within autonomous driving modalities, necessitate real-time responsiveness. However, the integration of real-time data streaming and processing functionalities into the vehicle software of electric vehicles is not yet fully realized, with the majority of big data analytics currently being conducted in cloud-based environments. This reliance on cloud computation highlights a significant area for advancement in vehicle software, as the transition to real-time, onboard processing would substantially enhance the agility and reliability of autonomous driving systems.
6. Hardware Miniaturization in Evolutionary Computing: The bulk of vehicle hardware, which has its origins in computer systems or industrial computer systems, does not align with the miniaturization requirements essential for components in evolutionary computing. The large-scale hardware is often incompatible with the compact design necessary for the sophisticated and space-efficient computing solutions that are increasingly demanded in the context of advanced vehicular systems. This discrepancy underscores the need for innovative hardware development that can accommodate the shrinking form factors while maintaining, or even enhancing, the computational prowess required for the next-generation of electric vehicles.
7. Deep Learning Adaptability in Dynamic Environments: To date, deep learning algorithms have struggled to readily accommodate minor variations within application environments. Confronted with diverse models and a spectrum of application scenarios, it is often imperative to retrain the autonomous driving system models, a necessity that extends to Large Language Model (LLM) algorithms as well. This challenge underscores the pressing need for enhanced algorithmic flexibility and adaptive capabilities within the autonomous driving domain, as the ability to swiftly adjust to environmental nuances is critical for the safe and efficient operation of autonomous vehicles.
8. Envisioning Future Electric Vehicles with Embedded AI Agents: In the forthcoming era of electric vehicles, it is conceivable that hundreds or even thousands of AI agents could be embedded within the vehicle’s architecture, with the capacity for remote upgrading via OTA technology. As depicted in Figure 3, each AI agent would be tasked with the execution of specific duties, thereby partitioning complex tasks into more manageable components. Currently, the integration of AI agents and LLM is in its nascent stage (Yang et al., 2024). There remains a significant developmental journey ahead before these advanced computational entities can be effectively harnessed in on-vehicle applications, necessitating further research and innovation to bridge the existing gaps and realize the full potential of such technology in the context of autonomous and intelligent vehicles.
9. The IOV as a Collaborative Evolutionary Technology: The IoV, which serves as a collaborative evolution technology for electric vehicles, is currently in its nascent stages with respect to both industrial deployment and technological sophistication. The current evolutionary capacity of electric vehicles is predominantly confined to the individual unit’s development. However, the full realization of electric vehicles as complex multi-robot systems is contingent upon the IoV to fully manifest its substantial potential. This underscores the necessity for further advancements in IoV technology to enable a paradigm shift from isolated vehicle evolution to a collaborative and interconnected vehicular ecosystem.
10. Redesigning Mechanisms for Unstructured Environments: In contexts lacking structure, there is a critical need to thoroughly redesign numerous planning and decision-making execution mechanisms. Advances in autonomous driving technology are instrumental in tackling these challenges, offering innovative solutions to enhance the adaptability and responsiveness of electric vehicles in such environments.
11. Innovation in Continuous Evolutionary Capabilities: The pursuit of innovation in the continuous evolution capabilities of electric vehicles demands substantial financial investment and incurs significant expenses, which in turn impact industrial applications. Within the paradigm of SDV and AIDV, the proportion of vehicle research and development (R&D) costs allocated to software, computing power, and automotive electronics is escalating. This trend underscores the need for a strategic reallocation of resources within the automotive sector, prioritizing the development of advanced software and hardware solutions that are essential for the next generation of vehicles. The financial implications of these investments must be carefully managed to maintain the competitiveness of the industry while ensuring the delivery of cutting-edge, secure, and user-centric vehicular technologies (Deloitte, 2024).

In the progression of EVs toward ERs, several key themes have emerged. These include the paradigm shift from conventional to SDV and AIDV, the challenges and potential solutions associated with OTA updates, and the imperative for robust, miniaturized hardware to facilitate complex software integrations. Additionally, the significance of real-time processing and data analytics for autonomous driving capabilities has been highlighted, alongside the financial and security considerations inherent in these technological innovations. The discourse has underscored the necessity for continuous research and development to confront these multifaceted challenges, thereby ensuring the sustainable and secure advancement of EV technology.

## 6 Discussion

The convergence of mature technologies such as cloud computing, edge computing, big data analytics, 5G communications, and EEA forms the bedrock upon which the rapid evolution of EVs is built. This evolution has steered EVs toward the transformation into sophisticated evolutionary robots, with the IoV technology propelling the transition from single-robot to multi-robot systems.

Technologies including OTA updates and pre-embedded hardware, while not fully mature, have been implemented on a large scale and offer substantial potential for the robotic evolution of EVs. The advancement of autonomous driving systems has significantly enhanced the intelligence level of EVs, endowing them with characteristics reminiscent of sentient robots. Notably, there is a striking resemblance between the architecture of autonomous driving systems and the recently emerging embodied intelligent systems, suggesting that the design objectives of these systems are highly aligned. This alignment is expected to result in systems with similar functionalities, with the distinction that the hardware of EVs is not anthropomorphic.

LLMs, prompt engineering, and AI agents are all indicative of the path towards Artificial General Intelligence (AGI). The realization of AGI is poised to dissolve the boundaries between EVs and multi-robot systems, leading to an indistinguishable convergence of the two concepts. LLM technology facilitates seamless multilingual and multi-turn dialogue mechanisms, enhancing the interactivity and emotional appeal of EVs to humans. Despite LLMs not yet reaching their full potential, their implementation and deployment in real-world applications are anticipated to proceed at an exceedingly rapid pace.

AI agents, defined as intelligent entities capable of perceiving the environment, making decisions, and executing actions, possess attributes such as memory, logical analysis, problem-solving, and issue integration capabilities. These features render AI agents highly adaptable for deployment in EVs. The ideal AI agents are envisioned to possess capabilities including autonomous thinking, tool utilization, memory, and multimodal comprehension. However, the current reliance on LLM models for independent thought and autonomous planning is not yet sufficient to guarantee the effectiveness and success rate of AI agents, with significant gaps persisting in these areas.

As EVs evolve toward becoming evolutionary multi-robot systems, numerous technical and policy challenges remain. Yet, in tandem with the ongoing maturation of the EV market, the forthcoming future is expected to yield a plethora of innovative technologies and applications in this burgeoning field of convergence.

## Data Availability

The original contributions presented in the study are included in the article/supplementary material, further inquiries can be directed to the corresponding author.

## Glossary

ADS: Autonomous driving system
AGI: Artificial General Intelligence
AI: Artificial Intelligence
AIDV: AI-Defined Vehicles
CTO: Chief Technology Officer
DCU: Domain Control Unit
ECU: Electronic Control Unit
EEA: Electrical/Electronic Architecture
ER: Evolutionary Robots
EV: Electric Vehicles
FOTA: Firmware-Over-The-Air
HW: Hardware
IoT: Internet of Things
IoV: Internet of Vehicles
LLM: Large Language Model
OEM: Original Equipment Manufacturer
OTA: Over-The-Air
QoS: Quality of Service
R&D: Research and Development
RT: RealTime
SDV: Software-Defined Vehicles
SOTA: Software-Over-The-Air
SW: Software
V2V: Vehicle-to-Vehicle
V2X: Vehicle-to-Everything
